# Tunable Cell-Adhesive Surfaces by Surface-Initiated Photoinduced Electron-Transfer-Reversible Addition–Fragmentation Chain-Transfer Polymerization

**DOI:** 10.1021/acs.langmuir.3c02604

**Published:** 2024-02-08

**Authors:** Andriy R. Kuzmyn, Tanja G. Ypma, Han Zuilhof

**Affiliations:** † Laboratory of Organic Chemistry, Wageningen University & Research, Stippeneng 4, 6708 WE Wageningen, The Netherlands; ‡ Lumicks BV, Paalbergweg 3, 1105 AG Amsterdam, The Netherlands; § School of Pharmaceutical Sciences and Technology, Tianjin University, 92 Weijin Road, 300072 Tianjin, China

## Abstract

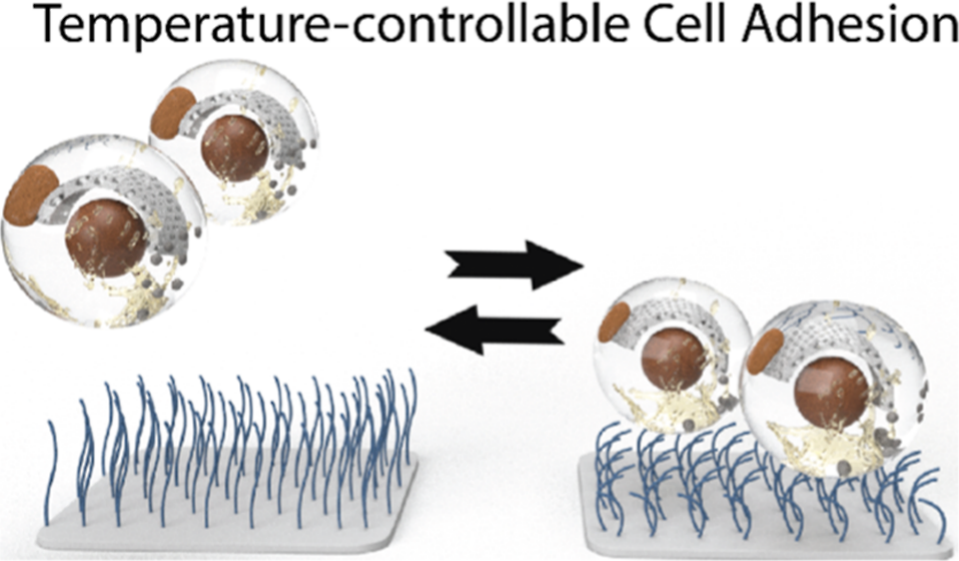

Cell adhesion involves many interactions between various molecules on the cell membrane (receptors, coreceptors, integrins, etc.) and surfaces or other cells. Cell adhesion plays a crucial role in the analysis of immune response, cancer treatment, tissue engineering, etc. Cell–cell adhesion can be quantified by measuring cell avidity, which defines the total interaction strength of the live cell binding. Typically, those investigations use tailor-made, reusable chips or surfaces onto which cells are cultured to form a monolayer to which other cells can bind. Cell avidity can then be measured by applying a force and quantifying cell–cell bond ruptures. The subsequent cleaning and reactivation of such biochip and biointeractive surfaces often require repeated etching, leading to device damage. Furthermore, it is often of great interest to harvest the cells that remain bound at the end of an avidity experiment for further analysis or use. It is, therefore, advantageous to pursue coating methods that allow tunable cell adhesion. This work presents temperature-switchable poly(di(ethylene glycol) methyl ether methacrylate) brush-based cell-interactive coatings produced by surface-initiated photoinduced electron-transfer reversible addition–fragmentation chain-transfer polymerization. The temperature switch of these brushes was explored by using a quartz crystal microbalance with dissipation monitoring, chemical composition, and physicochemical properties by atom force microscopy, X-ray photoelectron spectroscopy, single-molecule force spectroscopy, and ellipsometry.

## Introduction

For the development of novel immunotherapies, it is of crucial importance to clarify the binding strength between the disease-causing cells (often: cancer cells) and the “curing” effector cells (e.g., T-CAR cells).^1,2^ This binding strength or avidity is proving to be an important parameter in selecting cell lines that are most effective in killing such cancer cells. As a result, devices have been developed to measure such cell–cell or cell–surface interactions accurately,^3^ such as instruments that use piezo-electric effects to induce acoustic forces to measure this.^4,5^ This technique allows measuring the strength of attachment up to 1 nN for hundreds of individual cells in parallel.^3^ These interactions are mediated by the molecules on the surface or on surface-bound cells with those on the surfaces of the cells of interest. Such cell–cell and cell–surface interactions are known to be crucial for the efficiency, differentiation, and spatial direction of the development of cell cultures and thus also for the creation of specific tissues. As a result, understanding cell–cell interactions is crucial for fields that are as widely differing as cancer treatment, tissue formation, tissue engineering, and biosensing.

Often, cell-interactive chip-based devices are reused, given the cost of, e.g., any microfluidic components. In this process, the cell adhesion layer must be removed after the measurement so as to be able to fully regenerate the chip surface for future use. This cleaning and reactivation of such chips typically require chemically harsh substances, as the (near-)complete removal of any biological material is needed for any future use. In addition, glass chips typically require reactivation to expose silanol groups before reapplication of a coating that promotes cell adhesion. However, such etching is hard given the small size of these microdevices, while the etching itself already strongly hampers the multiple use of the microfluidic chips, leading to both substantially increased costs and material waste.

A way to overcome this problem is by the use of coatings that allow for both effective cell adhesion and tunable cell release. Surfaces coated with poly(ethylene glycol) (PEG) are among the most commonly applied coatings for preventing bioadhesion.^6−8^ Numerous publications showed that those coatings display good protein repellence or protein antifouling properties.^9−11^ Thus, PEG-based coating materials are studied and applied to different biotechnological applications, such as blood-compatible materials,^8^ implants,^12^ and stealth carriers for either drug delivery^13^ or gene delivery systems.^14^ Previously, PEG-based random copolymers 2-(2-methoxyethoxy)ethyl methacrylate (MeO2MA) and oligo(ethylene glycol) methacrylate (OEGMA) have demonstrated thermoresponsive and controlled cell adhesion properties.^15^ Surfaces modified with responsive polymers allow for the dynamic altering of their physicochemical properties in response to changes in applied stimuli and the covalent attachment of such polymer-coated surfaces with the required stability. Typically, those coatings are prepared using surface-initiated polymer brush-forming methods,^16^ specifically surface-initiated atom-transfer radical polymerization.^17^ This technique typically requires heavy metal and oxygen-free environments, which often hamper further application or scaling up of the approaches. New approaches might reduce this need.^7,18−20^

As a consequence, novel methods utilizing reversible addition–fragmentation chain transfer (RAFT) have emerged.^21^ In particular, the development of photoinduced electron-transfer-RAFT (PET-RAFT) polymerizations allow for the synthesis of polymer brushes even in the presence of oxygen, without the need for heavy metal catalysts.^7,19,20,22−24^ Additionally, this technique’s light-triggered nature facilitates the fabrication of hierarchical, patterned structures.^7,20^ The mild conditions of PET-RAFT techniques, utilizing dyes like Eosin Y and triethanolamine as catalysts in an aqueous environment, have been used to synthesize polymers from both cells and DNA.^25,26^ Recent reports have highlighted the feasibility of generating polymer brush coatings via surface-initiated photoinduced electron-transfer reversible addition–fragmentation chain-transfer polymerization (SI-PET-RAFT) on silicon and gold surfaces.^7,24,27^ These coatings have been employed to create antifouling,^7^ bioactive,^24^ and antiviral surfaces.^22^ Furthermore, they have been applied under flow conditions to further control the properties of the polymer brush including strongly improved linearity of growth and increased thicknesses.^19^ As of our current understanding, SI-PET-RAFT techniques have not yet been utilized for the development of switchable and cell-interactive surfaces.

In this paper, we report on the construction of thermoresponsive polymer brush coatings based on poly(di(ethylene glycol) methyl ether methacrylate) synthesized by applying mild metal-free SI-PET-RAFT polymerizations in water under ambient conditions. The resulting polymer brushes were subsequently characterized in terms of their composition and topology by using scanning ellipsometry, atom force microscopy (AFM), and X-ray photoelectron spectroscopy (XPS). The responsiveness was studied using both quartz crystal microbalances with dissipation monitoring (QCM-D) and single-molecule force spectroscopy (SMFS). Finally, the controlled cell adhesion onto and release from those coatings were tested in a flow chip with light microscopy using NALM6 cells. NALM6 is a human B cell precursor leukemia cell line, which has among many other things, been used as a model for optimizing CAR-T targeting cancer cells.^28,29^ Since in such studies cell adhesion is crucial, we reasoned that this cell line would be of particular relevance for this study.

## Materials and Methods

### Materials

Unless stated otherwise, all chemical reagents were utilized without undergoing additional purification. (3-Aminopropyl)triethoxysilane (APTES), 4-cyano-4-(phenylcarbonothioylthio)pentanoic acid *N*-succinimidyl ester (RAFT-NHS), acetone (99.5%), di(ethylene glycol) methyl ether methacrylate (MeO2MA), eosin Y (EY), ethanol (EtOH) (99.9%), dry tetrahydrofuran (99.9%), triethanolamine (TEOA), and triethylamine were acquired from Sigma-Aldrich (Merck). *N*-(2-Hydroxypropyl) methacrylamide (HPMA) was obtained from Poly Sciences, Inc. Quartz crystal microbalance chips were acquired Quantum Design GmbH. Glass substrates were acquired from Micronit. Silicon substrates were purchased from Siltronix. Milli-Q water was generated using a Milli-Q integral 3 system from Millipore in Molsheim, France.

#### Light Source

LEDs emitting at a peak intensity of 410 nm (Intelligent LED Solutions, product number: ILH-XO01-S410-SC211-WIR200) were employed, with the current adjusted to 700 mA as per manufacturer guidelines, resulting in a total radiometric power of 2.9 W. The measured light intensity of the halogen lamp was 3.5 μW·cm^–2^.

### Formation of APTES Layer on Oxide-Coated Silicon Surfaces

The formation of amino-terminated layers from the application of APTES onto silico-oxide substrates followed established procedures outlined in our prior publications.^7,19,22^

### Formation of RAFT Agent-Functionalized Monolayers

The RAFT-agent immobilization was conducted in accordance with previously published procedures.^7,19,22,24^

### SI-PET-RAFT Synthesis of Polymer Brushes

The polymerization was conducted according to a modification of a previously reported procedure.^7,19,22,24^ A solution containing the photocatalyst was prepared as follows: EY (25 mg, 39 μmol) and TEOA (160 mg, 1.6 mmol) were dissolved in 10 mL of Milli-Q water. Separately, the monomer MeO2MA (60 mg, 0.3 mmol) was dissolved in 1 mL of Milli-Q water, followed by the addition of 10 μL of the prepared stock solution. The resulting mixture was vortexed and applied to vials containing surfaces coated with an immobilized RAFT agent. Polymerization was initiated immediately by exposing the vials to visible light emitted by a light-emitting diode light source for 10 min. The thickness of the polymerization solution atop the surfaces measured 2 mm, while within a glass-closed system, it corresponded to the flow channel height in the LUMICKS chip, approximately ∼100 μm. During these experiments, the substrates were positioned at a distance of 3–4 cm from the light source. Polymerization ceased upon turning off the light. Samples were then taken out of the solution, rinsed successively with Milli-Q water and ethanol, and dried using a stream of argon gas.

### X-ray Photoelectron Spectroscopy

XPS measurements were performed in accordance with established procedures outlined in our prior publications.^19^

### Static Water Contact Angle Measurements

The evaluation of modified surface wettability was conducted through automated static water contact angle measurements, utilizing a Kruss DSA 100 goniometer following established procedures detailed in earlier publications.^7,22−24^

### Spectroscopic Ellipsometry

The thickness of the polymer brush layers was determined using the Accurion Nanofilm_ep4 Imaging Ellipsometer, following previously published techniques.^19^ Subsequently, the polymer brush layers were characterized using a Cauchy model with parameters *A* = 1.450 and *B* = 4500.

### Atomic Force Microscopy

Surface topography images using AFM were captured utilizing an Asylum Research MFP-3D Origin AFM (Oxford Instruments, United Kingdom), following established methodologies from our prior publications.^7,17,19,20,22,23^ The instrument operated in tapping mode, employing a silicon cantilever (AC240TS-R3, *k* = 1.3 N/m) with an approximate tip radius of ∼7 nm. Analysis and processing of the AFM topography images were performed using Gwyddion^30^ software.

### Quartz Crystal Microbalance with Dissipation Monitoring

QCM-D measurements were performed in accordance with techniques previously published.^24^

### Polymer Brush Switch Experiments

The borosilicate glass-coated QCM-D chips with immobilized polymer brushes according to the procedure described above were used in experiments. The temperature in the QCM-D chamber was changing from 18 to 40 °C with monitoring of change Δ*f*. The flow rate in the chamber was set at 6 μL·min^–1^. The change in the water content was used as an indication of the swelling or collapsing of the polymer brush.

### Aminolysis

The chain-end RAFT-agent aminolysis was conducted in accordance with previously published procedures.^31^

### Single-Molecule Force Spectroscopy

The SMFS measurements were conducted following a previously demonstrated methodology.^19^

### Cell Experiments

The cells used in the experiment were Nalm6 (ATCC). Square glass substrates (Micronit) and closed microfluidic LUMICKS chips were used in the experiment. A Nalm6 (ATCC) cell solution of >180 mln/mL concentration was prepared in serum-free medium (RPMI, Gibco). The LUMICKS chips were prepared by flushing phosphate-buffered saline, followed by serum-free medium into the channel. The cell solution was introduced into the LUMICKS chip channel by filling the chip reservoir with cell solution and pulling the syringe (3 mL, Thermo Fisher) with 200 μL to create a subtle flow. The cell solution was introduced on top of the Micronit chips in a 12-well plate by pipetting 2 mL of solution into the well. The cells were incubated at 37 °C for 30 min on the surface coatings. After the incubation, the cell adhesion was tested by a stability test. The stability test on the LUMICKS chips was done by flushing the channel with complete medium (RPMI + 10% Fetal Bovine Serum) by pulling the syringe with 200 μL for 5 s to rinse off unattached cells on the coating surface. The stability test on the square glass chips was done by aspirating and pipetting new complete medium into the 12-well plate. The monolayers were left at 37 °C for 2 h in complete medium. Then, the layers were allowed to cool down from 30 min to a temperature of 20 °C, followed by the stability test pulling the syringe with 500 μL for more than 10 s. Monolayer confluency after stability testing was done by brightfield imaging.

### Stability Test

Stability tests to test the cell adhesion on the surface coating were performed by introducing serum-free or complete cell medium (RMPI, Gibco) into the LUMICKS chip channel using a subtle flow created by pulling 200 μL on a syringe (3 mL, Thermo Scientific) for 5 s. The cell adhesion was checked with a bright-field microscope to determine the confluency before and after the stability test.

## Results and Discussion

**Scheme 1:**
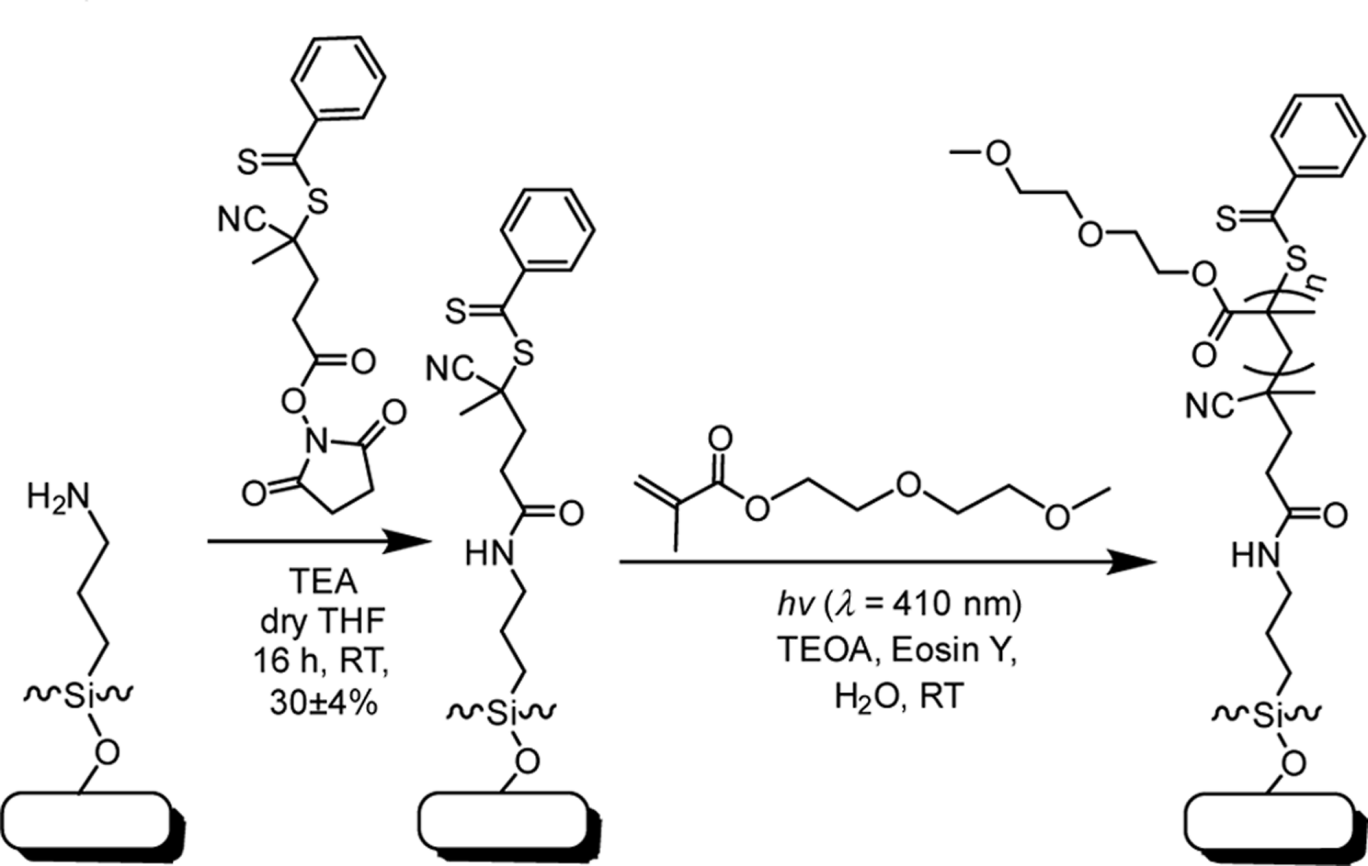
General Scheme of Synthesis of Poly(MeO2MA) Brush-Based Coating by SI-PET-RAFT

The creation of switchable polymer brush surfaces commenced with four sequential steps, beginning from untreated silicon surfaces (Scheme 1). Oxide-coated Si or borosilicate glass surfaces were prepared and activated using an oxygen plasma and subsequently immediately immersed in a freshly prepared solution of APTES (2 mg·mL^–1^ in absolute ethanol at RT for 16 h). The resulting amine-terminated surfaces were reacted with 4-cyano-4-(phenylcarbonothioylthio)pentanoic acid *N*-succinimidyl ester (RAFT-NHS) yielding a RAFT agent-functionalized monolayer. From the RAFT agent-coated surfaces, switchable polymer brushes based on di(ethylene glycol) methyl ether methacrylate (MeO2MA) were grown using SI-PET-RAFT in the presence of EY and TEOA as catalysts. Polymerization was conducted for 10 min to achieve an average thickness of 15.4 ± 1.5 nm, as determined by ellipsometry.

**Figure 1:**
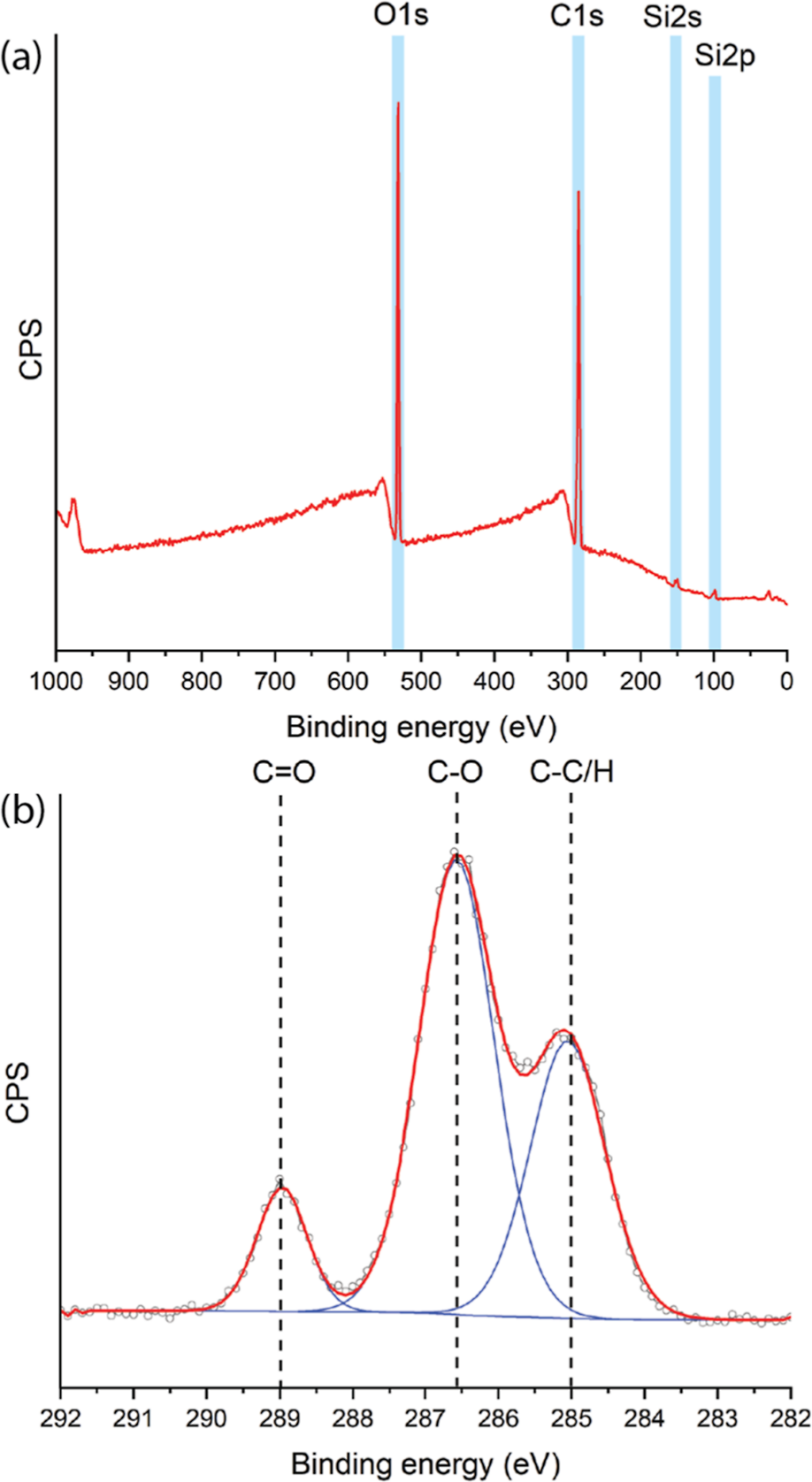
XPS Characterization of poly(MeO2MA) brushes synthesized by SI-PET-RAFT. (a) Wide-scan spectrum. (b) Narrow-scan C 1s spectrum.

The chemical composition of these poly(MeO2MA) brushes was investigated using XPS. The XPS wide-scan spectrum of a poly(MeO2MA) layer with a thickness of 15 nm, as determined by ellipsometry, showed two main peaks for O 1s and C 1s in an average ratio of 1.0:2.7 (Figure 1a). The XPS narrow-scan spectrum of the C 1s region shows three main peaks for [**C**–C/H], [**C**–O], and [O–**C**=O] in an average ratio of 3.4:5.4:1.0 (Figure 1b). The simulated C1 XPS spectrum, obtained using standard DFT methods,^32,33^ gives three peaks (in the theoretically expected ratio 3:5:1) for [C–C/H]/[C–O]/[O–C=O] (see Figure S1) and displays good agreement with the experimental data. The static water contact angle of those coatings is 64 ± 2°. The AFM topography images of brush-coated surfaces revealed highly homogeneous layers with a roughness of *R*_q_ = 3.9 ± 1.1 nm (see Figure S2). The molecular weight and grafting density of thus obtained poly(MeO2MA) brushes were determined by SMFS,^31,34^ as recently applied by us^35^ to determine the physicochemical properties of brushes made by SI-PET-RAFT. The contour length of a poly(MeO2MA) brush with a dry thickness of 15 ± 1.5 nm (by ellipsometry) was determined to be 75 ± 4 nm, and the corresponding molecular weight of the polymer is then derived to be 51.5 ± 2.4 × 10^3^ g·mol^–1^ (see Figure S3). The grafting density σ of the polymer chains can then be determined to be 0.19 ± 0.01 polymer chains per nm^–2^ using the equation σ = *h·*ρ*·N*_A_/*M*_n_, where *h* = dry thickness of the brush as determined by ellipsometry, ρ = bulk density of poly(MeO2MA), and taken to be 1.05 g·cm^–3^,^35^ and *N*_A_ = Avogadro’s constant. This value is appreciably higher than the values recently reported by us for the analogous polymer brush with 5–6 EO units (rather than the 2 EO units in the current study), namely, 0.07 ± 0.01 and 0.08 ± 0.01 polymer chains per square nanometers. We attribute this higher packing density to the smaller side chains in the current polymer brushes, which allow a tighter packing.

**Figure 2:**
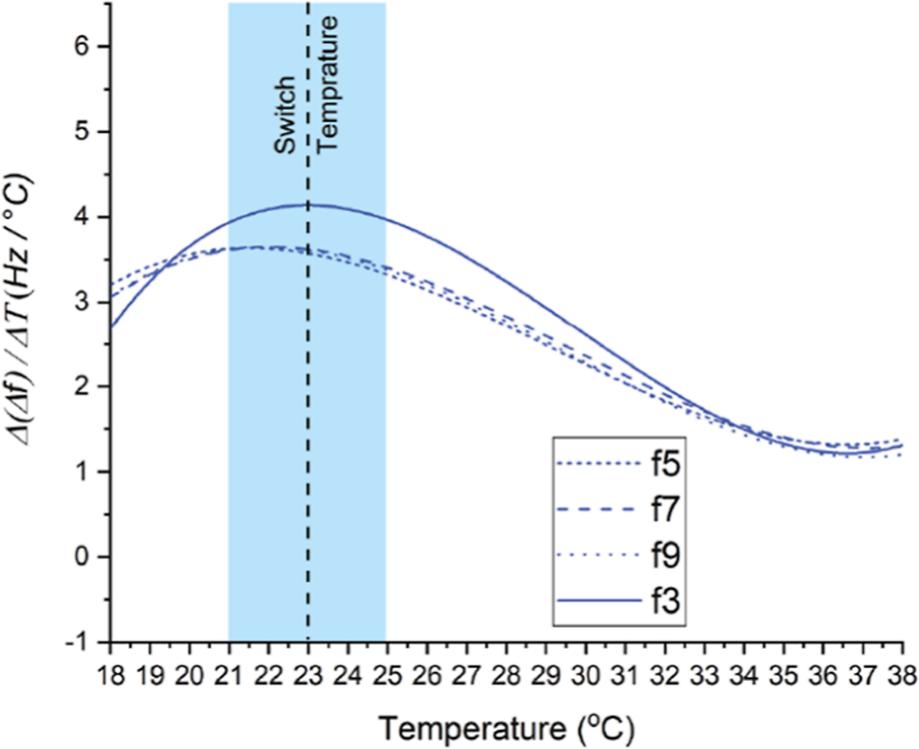
QCM-D measurements of poly(MeO2MA) coatings obtained by SI-PET-RAFT. The graph depicts the deferential of change of frequency (Δ*f*) and change of temperature (Δ*T*) acquired at different harmonic overtones third (f3) (15 MHz), fifth (f5) (25 MHz), seventh (f7) (35 MHz), and ninth (f9) (45 MHz).

Temperature-switchable polymers exhibit a temperature-dependent change in solubility, which is typically investigated in aqueous solutions. At the temperature of switching, phase transitions induce a conformational change of the polymer structure, corresponding to a soluble ⇆ insoluble transition. Polymer chains that are dissolved adopt a swollen and randomized coil conformation. Upon desolvation at increased temperatures, the polymers collapse to form globule-like structures.^36^ The temperature of the solvation-induced switch of these polymer brushes was determined using a QCM-D (Figures 2 and S4). The QCM-D measurements were performed between 18 and 38 °C. Borosilicate glass-coated QCM-D sensors were first modified with poly(MEO2MA) brushes according to the procedures outlined above. Additionally, reference experiments were performed using pristine borosilicate glass QCM-D sensors.

In a typical experiment, the QCM-D chamber was kept in a water environment, the temperature of which was gradually increased from 18 to 38 °C and—subsequently and at the same speed—decreased back to 18 °C, while the changes in the resonance frequencies (Δ*f*) of the surfaces were measured continuously. The switching temperature of polymer brushes was determined from the differential of the change of frequency (Δ*f*) versus the change of temperature (Δ*T*). The highest point of this curve indicates the temperature switch and is related to the maximum sensitivity of the amount of water released or readsorbed by polymer brushes. This is ultimately connected to the surface mass and change of frequency (Δ*f*) in the QCM-D system. In this manner, from a series of temperature-dependent QCM-D measurements, we determined the switching temperature of poly(MeO2MA) brushes in the thickness range of 15.4 ± 1.5 nm to be 23 °C.

As such switching is of interest to any biomedical device only when highly reproducible, we performed these experiments for 100 cycles of heating and cooling. This also provides detailed information on the stability of these polymer brushes in aqueous conditions and their water uptake (see Figure S5). The experiment displayed unchanged temperature-induced switching for 100 cycles, implying that no mass loss was observed during QCM-D measurements and that the functional stability of these polymer brushes is high.

**Figure 3:**
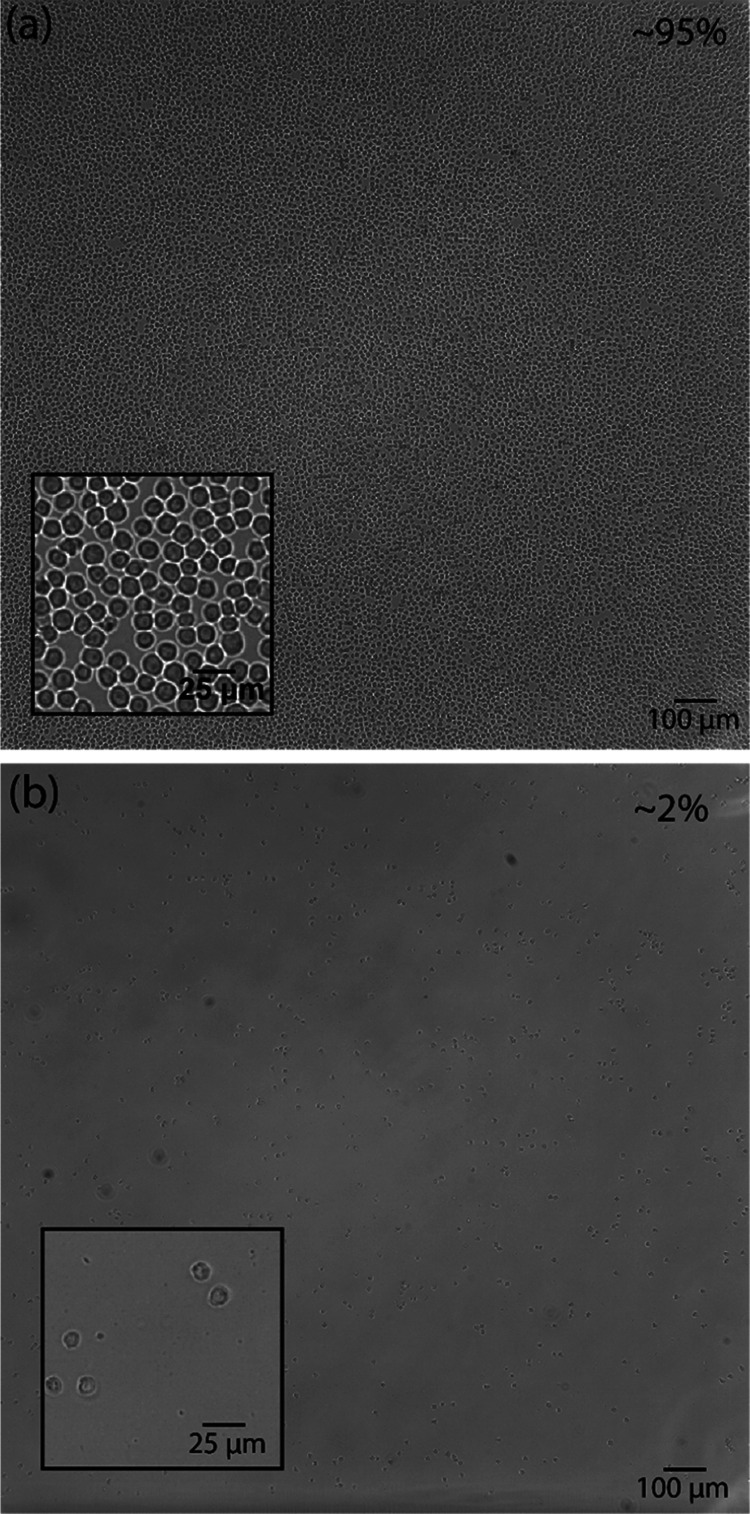
Cell adhesion. (a) Nalm6 cell layer at 37 °C on the poly(MeO2MA) brush-based coatings after the stability test. (b) Nalm6 cells monolayer after cooling to 20 °C and introduction of the flow. The cell confluency is indicated in the upper right corner of the image.

Next, we investigated cell adhesion on poly(MeO2MA) brush-based coatings using Nalm6 cells. The adhesion of the cells was observed in a LUMICKS z-Movi microfluidic glass chip with integrated piezo transducers. This setup allows cell avidity measurements by bright-field microscopy, via the use of an acoustic force field while maintaining samples under physiological conditions in the closed system of a flow-through chip. Figure 3a demonstrates the formation of a monolayer of cells after incubation at 37 °C for 2 h and the performance of an adhesion stability flow test. Following the successful stability test, these cell-covered chips were cooled to room temperature (20 °C), at which temperature the polymer brush was expected to undergo the phase transition described above and the cell medium was again gently pushed in the chamber. The result of this action is demonstrated in Figure 3b: switching of the temperature leads to a near-complete removal of cells and thus to easy recycling of the biofunctionalized device. This result further confirms the great thermal switching properties of poly(MeO2MA) brushes in interactions with cells (Figure S5). Specifically, the easy thermally triggered release of the cells from poly(MeO2MA) just by the introduction of a cold flow opens routes for the application of those coatings in a wide range of different biointeractive devices. Further studies using a wider range of polymer brushes and various cell lines are currently underway in our laboratories.

## Conclusions

We applied the SI-PET-RAFT technique to create thermally switchable poly(MeO2MA) polymer brushes. This polymerization technique allows a simple and mass-manufactured approach to creating covalently bound polymer brushes. The polymer brushes were characterized with XPS, ellipsometer, AFM, and SMFS. The switching temperature of these poly(MeO2MA) brushes was determined at 23 °C. The brush coatings showed good chemical stability and highly reproducible thermal switching over 100 cycles of repeated heating and cooling. Finally, at this switching temperature, the adhesion and release of the Nalm6 cells on poly(MeO2MA) coatings were confirmed by bright-field microscopy, further showing the power of such brushes for a wide range of biomedical studies.

## Supporting Information Available

XPS simulation; AFM topography; SMFS measurement details; QCMD sensograms; and additional cell experimental data (PDF)
