# Supplementary material for: Tunable Cell-Adhesive Surfaces by Surface-Initiated Photoinduced Electron-Transfer-Reversible Addition–Fragmentation Chain-Transfer Polymerization
Source: Langmuir. 2024 Feb 8;40(7):3354–9. doi: 10.1021/acs.langmuir.3c02604 (PMC10883044; doi:10.1021/acs.langmuir.3c02604)
Supplement: Supplementary file 1 — la3c02604_si_001.pdf [file la3c02604_si_001.pdf]

Supporting Information for:

Tunable Cell-Adhesive Surfaces by Surface-Initiated Photoinduced Electron Transfer-Reversible Addition–Fragmentation Chain Transfer Polymerization

Andriy R. Kuzmyn,[1,*] Tanja G. Ypma[2] and Han Zuilhof[1,3,*]

1) Laboratory of Organic Chemistry, Wageningen University & Research, Stippeneng 4, 6708 WE Wageningen, The Netherlands. 2) Lumicks BV, Paalbergweg 3, 1105 AG Amsterdam, The Netherlands. 3) School of Pharmaceutical Sciences and Technology, Tianjin University, 92 Weijin Road, Tianjin, 300072, China.

Table of content

XPS characterization S2

AFM topography S3

SMFS measurements S3

QCM-D measurements S4

Additional cell experiment images S6

Corresponding authors

andriy.kuzmyn@gmail.com ; Han.Zuilhof@wur.nl

XPS Simulations

	1	2	3	4	5	6	7	8	9	10	11	12	13
Natural Atomic Orbitals Occupancy Energy	9.90261	9.92659	10.05136	18.83705	9.89649	18.7083	9.97315	9.97	18.807	9.96421	9.96181	18.8044	9.95882
Binding energy (eV)	285.07463	285.757698	289.311756	539.57106	284.9003	535.9036	287.084	286.99	538.71	286.829	286.7609	538.64	286.6758

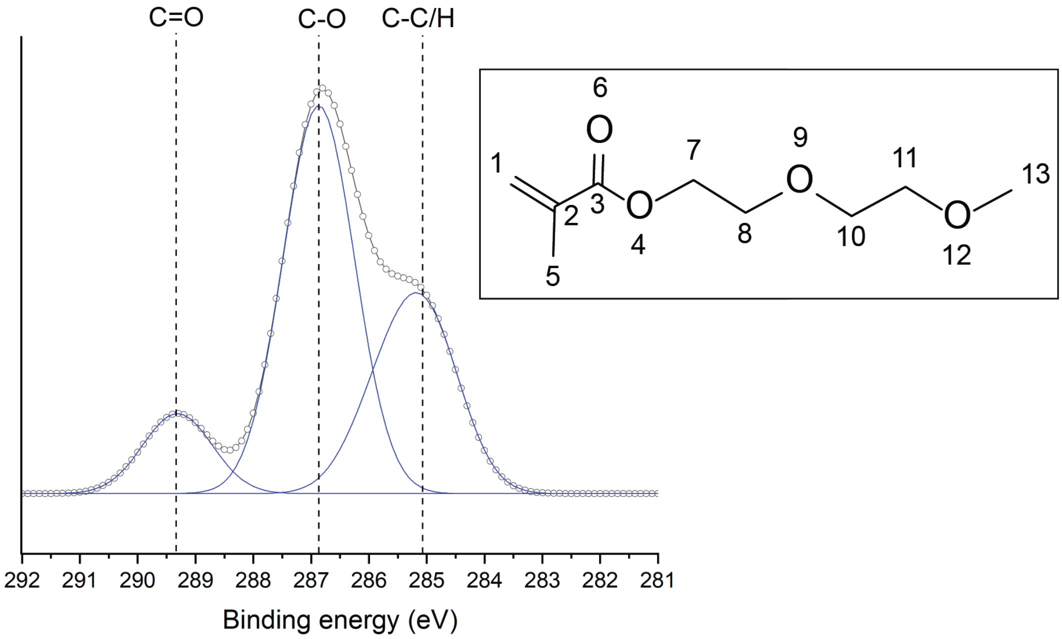

Figure S1. Simulated C1s XPS spectrum of the MeOMA based on the core orbital energy levels obtained by DFT calculations.

AFM topography

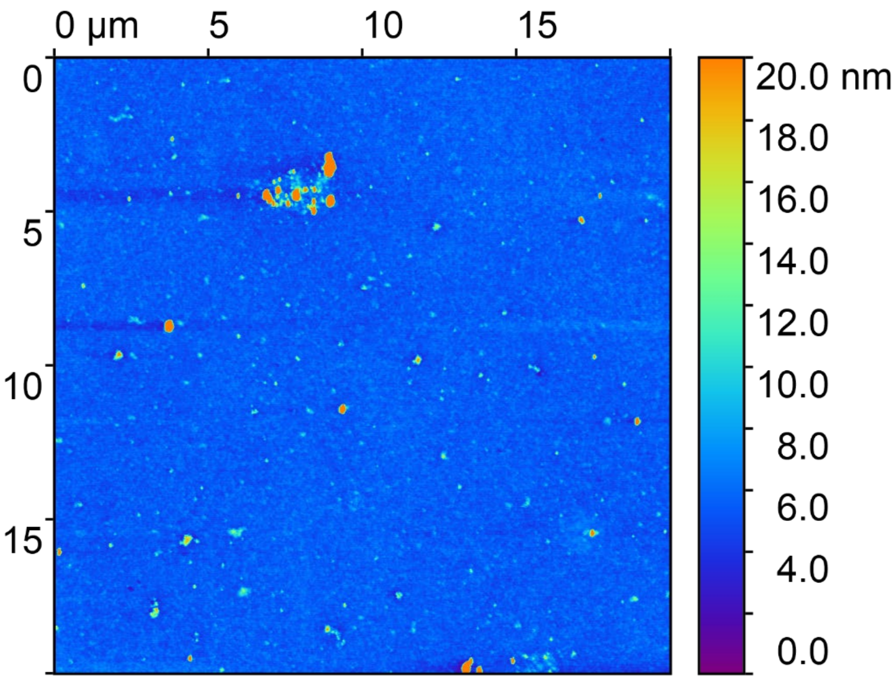

Figure S2. Representative AFM topography image of poly(MeOMA) synthesized by SI-PET-RAFT, polymerization time 10 min, thickness 15.4 nm.

SMFS measurements

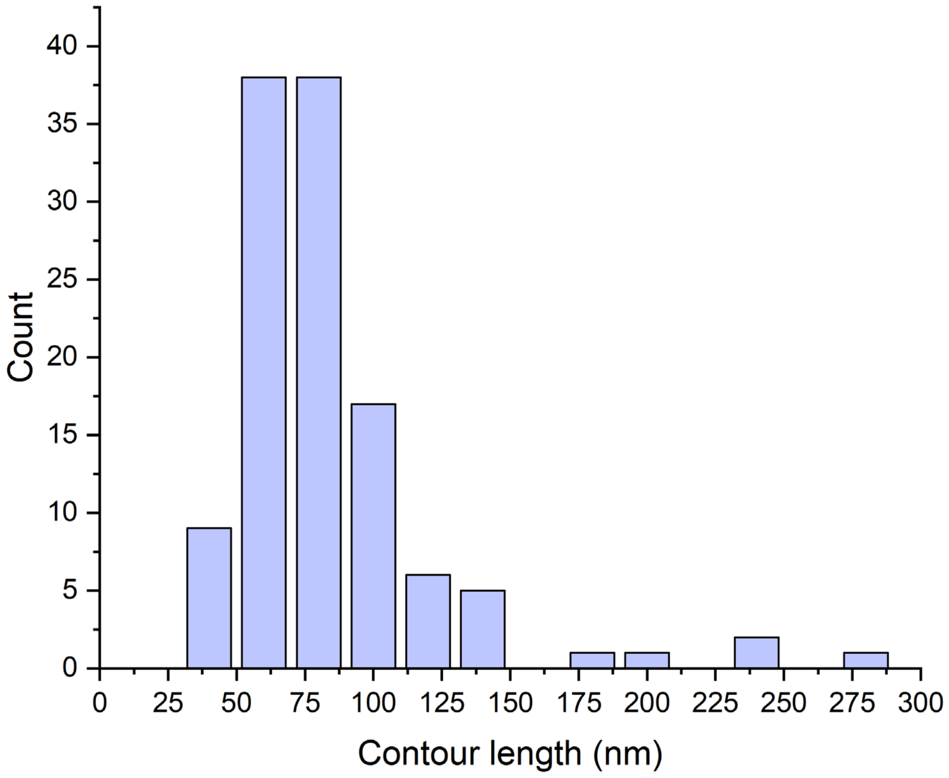

Figure S3. Distribution of contour lengths obtained from the fits to the wormlike-chain model as measured on thiol-terminated poly(MeOMA) brushes synthesized by SI-PET-RAFT, polymerization time 10 min, thickness 15.4 nm.

QCMD measurements

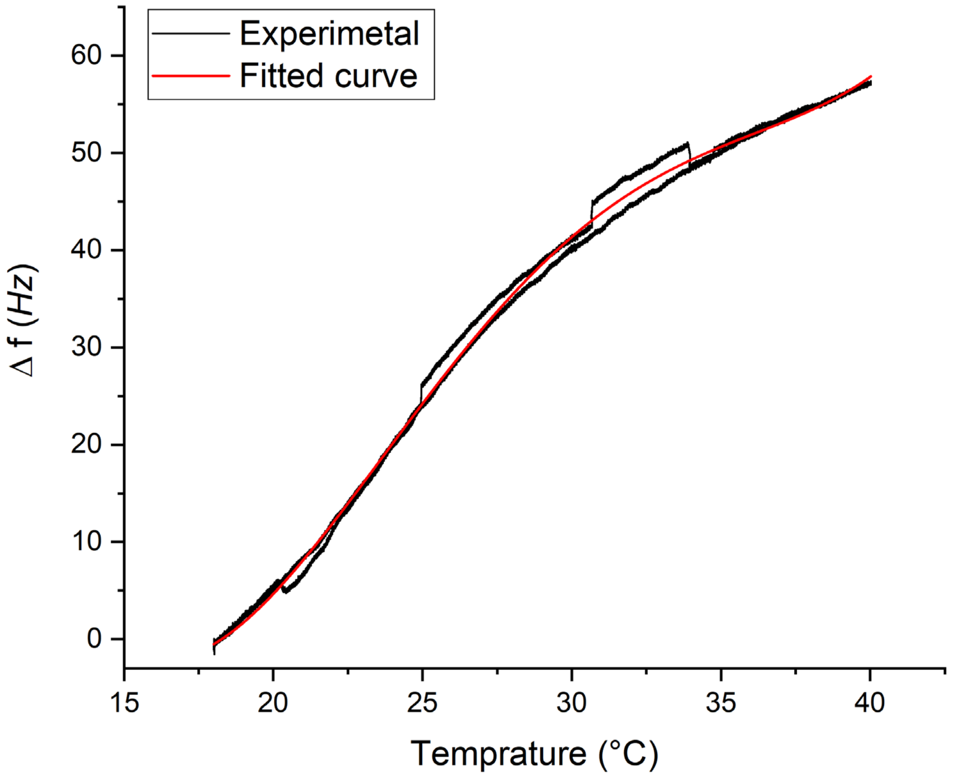

Figure S4. QCM-D data shows the frequency changes versus temperature changes with an increase of the temperature from 16 °C to 40 °C decrease of the temperature from 40 °C to 16 °C in the red line is the fitted average curve of both plots. This curve is further used for calculations of derivatives.

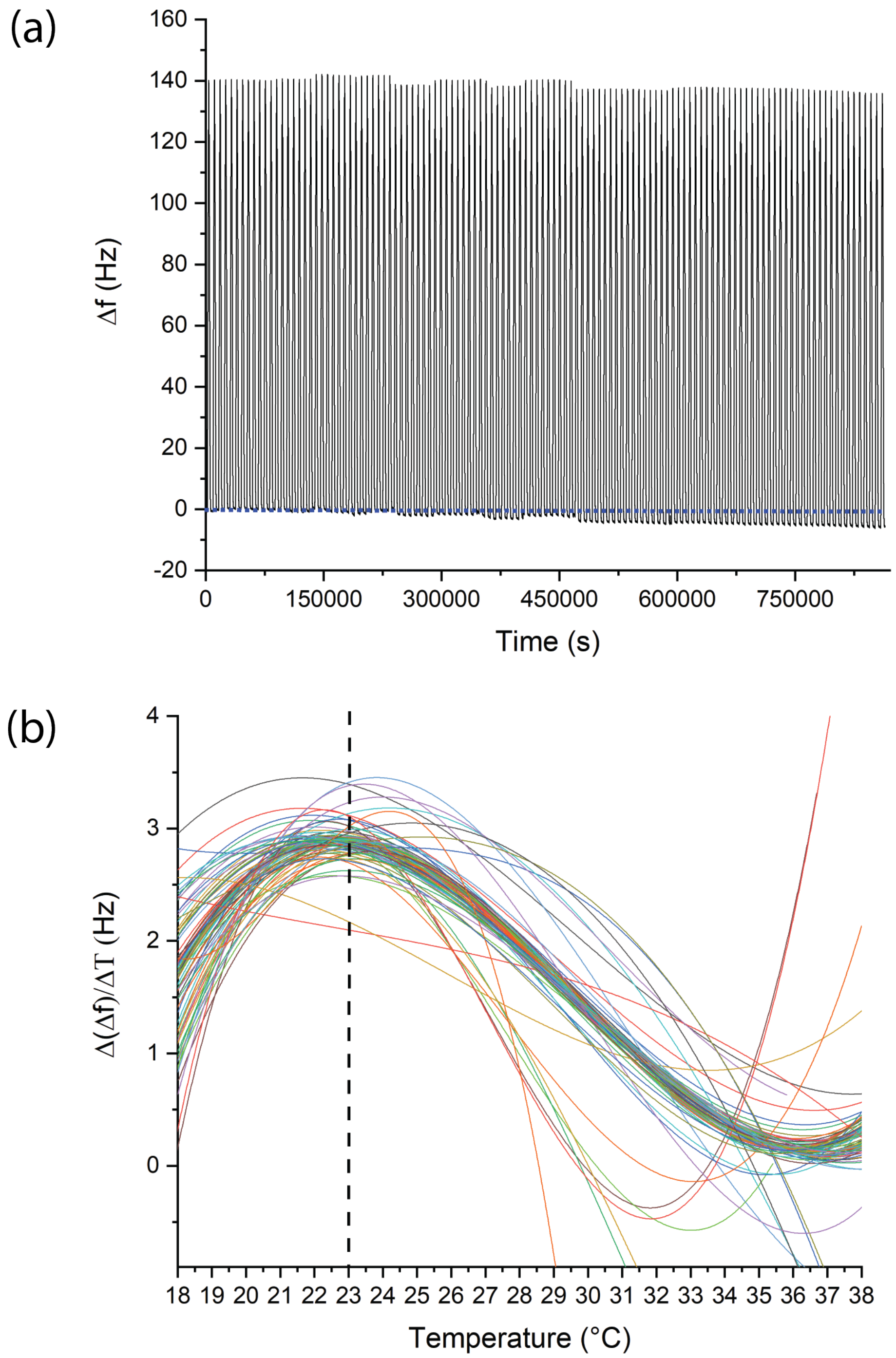

Figure S5. QCM-D data shows the mass changes due to changes in water content in the poly(MeOMA) brush during 100 heating and cooling cycles from 18 to 38°C. (a) QCM-D plot of frequency (Δf) (b) the deferential of change of frequency (Δf) and change of temperature (ΔT) acquired at (f3)(15 MHz) for 100 cycles.

Additional cell experiment images

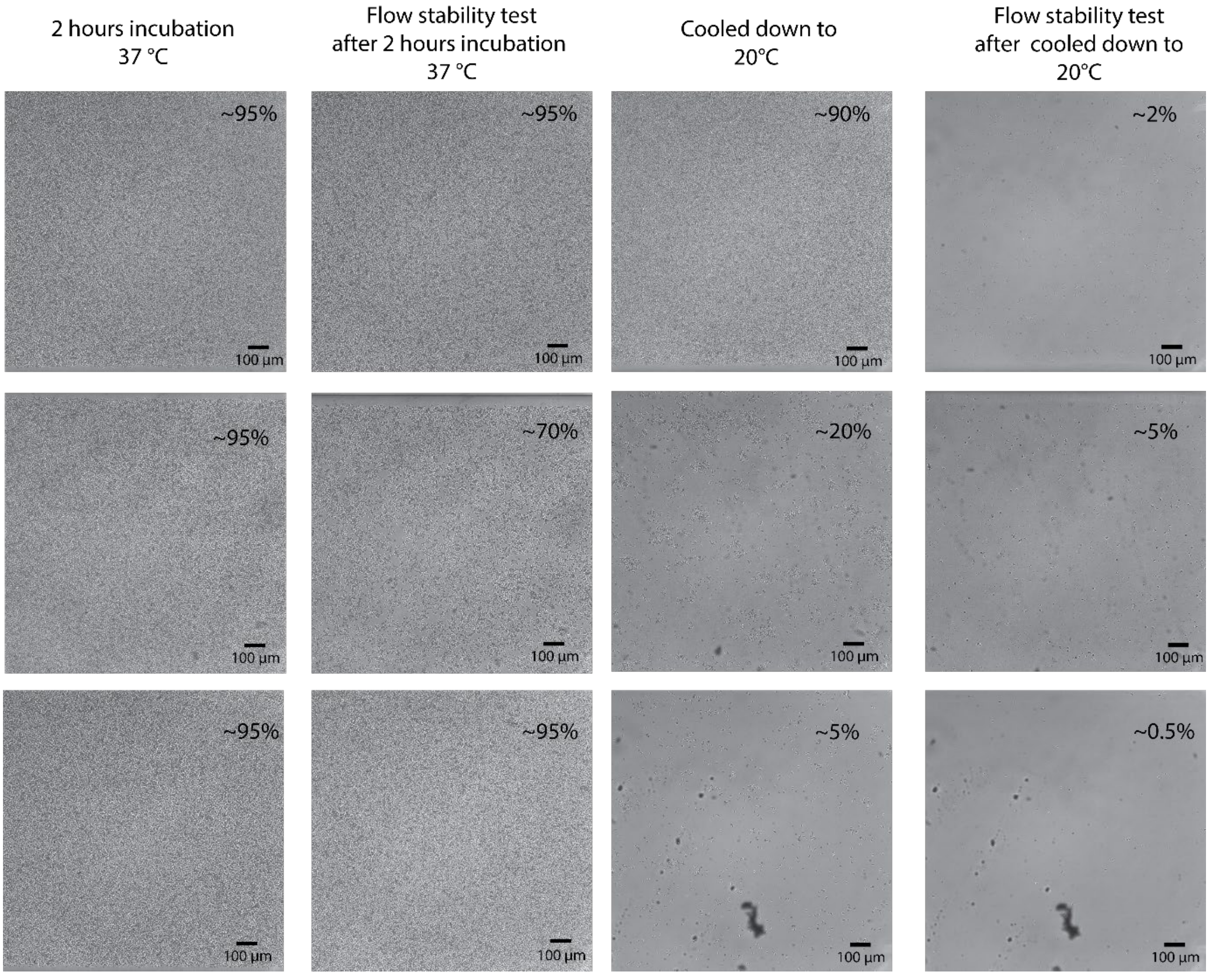

Figure S6. Monolayers of Nalm6 cells on poly(MeOMA) coatings were incubated at 37 °C for 2 h, then the flow stability test was introduced at 37 °C, and further, the cell monolayer cooled down to 20 °C with further introduction flow at 20 °C. The cell confluency is indicated in upper right corner of the image.
